# Ambient Volatile Organic Compound Characterization, Source Apportionment, and Risk Assessment in Three Megacities of China in 2019

**DOI:** 10.3390/toxics11080651

**Published:** 2023-07-27

**Authors:** Zhanshan Wang, Puzhen Zhang, Libo Pan, Yan Qian, Zhigang Li, Xiaoqian Li, Chen Guo, Xiaojing Zhu, Yuanyuan Xie, Yongjie Wei

**Affiliations:** 1 State Key Laboratory of Environmental Criteria and Risk Assessment, Chinese Research Academy of Environmental Sciences, Beijing 100012, China; 18701650609@163.com (Z.W.);; 2 Foreign Environmental Cooperation Centre, Ministry of Ecology and Environment, Beijing 100035, China

**Keywords:** VOCs, ozone, PSCF, source apportionment, EKMA, risk assessment

## Abstract

In order to illustrate pollution characterization, source apportionment, and risk assessment of VOCs in Beijing, Baoding, and Shanghai, field observations of CO, NO, NO_2_, O_3,_ and volatile organic compounds (VOCs) were conducted in 2019. Concentrations of VOCs were the highest in Beijing (105.4 ± 52.1 ppb), followed by Baoding (97.1 ± 47.5 ppb) and Shanghai (91.1 ± 41.3 ppb). Concentrations of VOCs were the highest in winter (120.3 ± 61.5 ppb) among the three seasons tested, followed by summer (98.1 + 50.8 ppb) and autumn (75.5 + 33.4 ppb). Alkenes were the most reactive VOC species in all cities, accounting for 56.0%, 53.7%, and 39.4% of ozone formation potential in Beijing, Baoding, and Shanghai, respectively. Alkenes and aromatics were the reactive species, particularly ethene, propene, 1,3,5-trimethylbenzene, and m/p-xylene. Vehicular exhaust was the principal source in all three cities, accounting for 27.0%, 30.4%, and 23.3% of VOCs in Beijing, Baoding, and Shanghai, respectively. Industrial manufacturing was the second largest source in Baoding (23.6%) and Shanghai (21.3%), and solvent utilization was the second largest source in Beijing (25.1%). The empirical kinetic modeling approach showed that O_3_ formation was limited by both VOCs and nitric oxides at Fangshan (the suburban site) and by VOCs at Xuhui (the urban site). Acrolein was the only substance with an average hazard quotient greater than 1, indicating significant non-carcinogenic risk. In Beijing, 1,2-dibromoethane had an R-value of 1.1 × 10^−4^ and posed a definite carcinogenic risk.

## 1. Introduction

In September 2013, the Chinese government implemented the Action Plan on Air Pollution Prevention and Control, resulting in significant reductions in ambient concentrations of CO, SO_2_, NO_2,_ and fine particulate levels nationwide [1,2,3]. However, O_3_ pollution has not decreased and appears to be worsening in China. The O_3_ concentration showed an increasing trend of 1–3 ppbv/y from 2013 to 2017 in eastern China [4]. Volatile organic compounds (VOCs) and nitrogen oxides (NOx) are the main precursors of O_3_. The relationship between O_3_ and its precursors is highly nonlinear due to the complex photochemical reactions that occur in the atmosphere [5,6,7]. Conditions for forming ground-level O_3_ can be divided into VOC-limited, NOx-limited, and both VOC- and NOx-limited [8]. In most developed areas of China, including the Yangtze River Delta, Jing-Jin-Ji, and Pearl River Delta regions, O_3_ formation is reported to be VOC-limited [9,10,11]. Thus, controlling VOC emissions is critical for reducing O_3_ pollution in China.

High concentrations of VOCs have adverse effects on public health by affecting the respiratory and cardiovascular systems [12,13,14,15,16]. Previous studies showed that cancer risk is greater in high-VOC-exposure areas than in clean areas [17,18]. Hazardous VOCs, including non-carcinogens and carcinogens, account for 20–40% of all non-methane VOCs in China [19]. Many VOC species, including benzene and 1,3-Butadiene, are classified as hazardous air pollutants by the United States Environmental Protection Agency (USEPA) and other international agencies [20,21,22,23].

The Beijing–Tianjin–Hebei (BTH) and Yangtze River Delta (YRD) regions are two of the largest urban agglomerations in China. Numerous studies of VOCs have been performed in BTH and YRD. Several studies have examined the general characteristics of VOCs and discussed their spatiotemporal variations [24,25,26,27,28]. Other studies have focused on the relative reactivity and ozone formation potential (OFP) of VOCs [29,30,31,32]. Several studies have aimed to reveal the health effects of VOCs [33,34,35,36,37,38]. Additionally, the emission inventory [39], regional transport [36,40,41,42], and source apportionment [26,43,44,45,46] of VOCs have been discussed.

Beijing, the capital of China, and Baoding, one of the most air-polluted cities, are both located in the BTH region. Shanghai is one of the most economically developed cities in the YRD and has relatively concentrated energy consumption and pollutant emissions. Most previous studies have been limited to a small number of sampling locations or a short sampling period. In this study, field observations of CO, NO, NO_2_, O_3,_ and VOCs were conducted in these three megacities. The main objectives of this study were to: (1) characterize the concentrations and spatiotemporal variations of VOCs; (2) discuss the regional transport and source apportionment of VOCs; (3) determine the roles of VOCs in ground-level O_3_ formation; (4) estimate the carcinogenic and non-carcinogenic risks of VOCs; and (5) identify the key hazardous VOCs in the three cities.

## 2. Methodology

### 2.1. Sampling Site and Period

**Figure 1:**
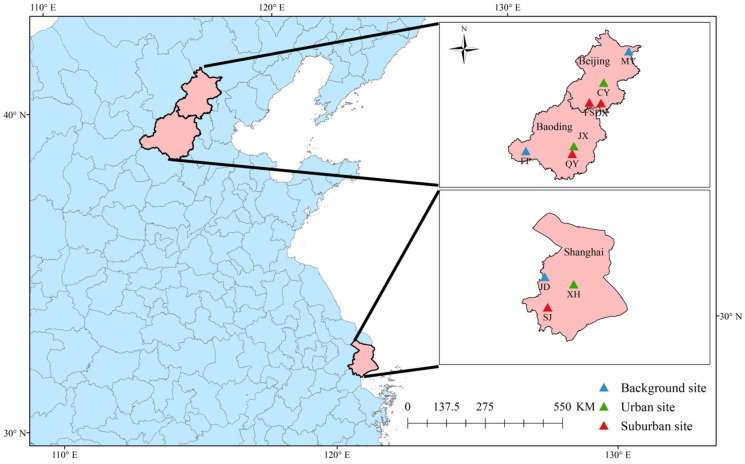
Sites sampled in this study. MY, FP, and JD are background sites. CY, JX, and XH are urban sites. FS, DX, QY, and SJ are suburban sites.

Ten sites in the three cities were selected for this study, among which four were in Beijing, three were in Baoding, and three were in Shanghai (Figure 1). The sampling sites in each city included a background site, an urban site, and a suburban site. A continuous 2-week period in each of the four seasons was selected. However, due to the COVID-19 crisis, sampling in the spring was terminated. Thus, the sampling periods were 15–28 August in the summer, 13–26 October in the autumn, and 18–31 December in the winter of 2019.

### 2.2. Sampling and Analysis

CO, NO, NO_2,_ and O_3_ were observed using a sensor-based air monitoring instrument developed by the Chinese Research Academy of Environmental Sciences. This instrument passed an intercomparison assessment with an instrument produced by TSI Corporations. The correlation coefficient was 0.92, and the two instruments’ relative error was 8%. VOCs were sampled at 16:00 at each site using a 3.2-L Summa canister (Entech Instruments Inc., Simi Valley, CA, USA). In total, 99 VOCs were observed, including 29 alkanes, 13 alkenes, 1 alkyne (acetylene), 16 aromatics, 32 haloalkanes, and 8 oxygenated VOCs (OVOCs). VOC samples were analyzed using the Agilent 5973N gas chromatography–mass selective detector flame ionization detector (Agilent Technologies, Santa Clara, CA, USA). A liquid nitrogen primary cryogenic trap with glass beads at −160 °C was used to trap VOCs. Then, the trap was heated to 10 °C, and target compounds were transferred to a secondary trap at −50 °C. Next, the VOCs were transferred using helium to a third trap at −170 °C. A DB-1 capillary column (60 m × 0.32 mm × 1.0 μm, Agilent Inc.) was used with helium as the carrier gas. Rigorous quality assurance and quality control procedures were employed. Periodic calibration was performed every 5 d. Calibration curve results for a given target species with less than 10% variation relative to the actual values were considered acceptable. Meteorological data were obtained from the China Meteorological Station Data Sharing Service System (http://cdc.cma.gov.cn/home.do, accessed on 5 January 2021).

### 2.3. Determination of the Ozone Formation Potential

The OFP can be used to characterize the maximum amount of O_3_ production possible from a given VOC species alone under optimal conditions. The key compounds responsible for O_3_ formation can be determined from the respective OFPs [38]. The OFPs are calculated based on the maximum incremental reactivity (MIR) of each individual species and given by the following equation:

OFP_i_ = VOCs_i_ × MIR_i_ (1)

where OFP_i_ is the OFP of VOC species i, VOCs_i_ is the concentration of VOC species i, and MIR_i_ is the O_3_ formation coefficient for VOC species i at the maximum incremental reactivity of O_3_ [47].

### 2.4. Positive Matrix Factorization Receptor Model

The sources of PM_2.5_ were analyzed using the positive matrix factorization (PMF) receptor model. First, the error associated with the chemical component weights of the receptor was determined. Then, the main sources of contamination and their contribution ratios were determined using the least squares method. PMF is a type of multivariate factor analysis in which a mathematical method decomposes matrix X containing sample data for a given species into two matrices: factor contributions (G) and factor spectra (F). This method does not require the input of a source spectrum and ensures that the decomposition factor contribution (G) and factor spectrum (F) are non-negative [48]. The following formula represents the matrix X:

(2) $$x_{ij}=\sum_{k=1}^{p}g_{ik}f_{kj}+e_{ij}$$

where $x_{\mathrm{ij}}$ is the concentration of species j in sample i, p is the number of factors, $g_{\mathrm{ik}}$ is the contribution of factor k to sample i, $f_{\mathrm{kj}}$ is the contribution of factor k to species j, and $e_{\mathrm{ij}}$ is the error of species j in sample i.

The uncertainty of a sample was calculated from the error fraction and the method detection limit (MDL). If the concentration was unknown, it was set to 1/2 of the geometric mean value. If the concentration was below the MDL, it was set to 1/2 of the MDL, and the uncertainty was set to 5/6 of the MDL. If the concentration was higher than the MDL, the uncertainty calculation was based on the error fraction as follows:

(3) $$Unc=\sqrt{{\left(Error\;Fraction\times concentration\right)}^{2}+{\left(0.5\times MDL\right)}^{2}}$$

The PMF analysis depends on the objective function (Q) to minimize the residual and uncertainty, as shown in Equation (4). The calculation of Q_exp_ is shown in Equation (5).

(4) $$Q=\sum_{i=1}^{n}\sum_{j=1}^{m}{\left[\frac{x_{ij}-\sum_{k=1}^{p}g_{ik}\times f_{kj}}{u_{ij}}\right]}^{2}\;$$

(5) $$Q_{exp}=n\times m-p\times \left(n+m\right)$$

where n and m are the numbers of species and samples, respectively, and u_ij_ is the uncertainty of the jth species in the ith sample.

Several aspects were considered to define a reasonable result (Liu et al., 2021) [22]: (1) the value of Q/Q_exp_ from PMF should be close to one; (2) the change rate of Q/Q_exp_ should be stable; and (3) the explored factors should be physically plausible and interpretable. Ultimately, a five-factor solution was determined in this study. The Q/Q_exp_ values were 1.3, 1.4, and 1.4 for Beijing, Baoding, and Shanghai, respectively.

### 2.5. Potential Source Contribution Function

The potential source contribution function (PSCF) was employed in this study using the software Meteoinfo (3.6.3) to identify the local and long-range transport pathways of VOCs. The PSCF is a backward-trajectory-based method that combines pollutant concentrations, reflecting the potential for this area to become a source of VOC pollution [49]. The PSCF is a position function defined by unit indexes i and j:

(6) $${PSCF}_{ij}=\frac{m_{ij}}{n_{ij}}W_{ij}$$

where n_ij_ is the number of trajectory endpoints that fall within the ijth grid cell, m_ij_ is the number of endpoints corresponding to trajectories that exceed the threshold criterion at the receptor site [50], and ij is the grid cell. The arbitrary weighting function W_ij_ was applied to reduce the uncertainty caused by small values of n_ij_:

(7) $$W_{ij}=\left\{\begin{array}{c} 0.70\;\;3n_{ave}>n_{ij}\geq 1.5n_{ave}\\ 0.42\;\;1.5n_{ave}>n_{ij}\geq n_{ave}\\ 0.05\;\;n_{ave}>n_{ij} \end{array}\right.$$

where n_ave_ is the average value of the endpoints of the trajectory through all the grids. In this study, the n_ave_ was 1.33, 1.31, and 1.19 for Fangshan, Jiading, and Jingxiu sites, respectively. In this study, the 24-h backward trajectory was calculated at 1-h intervals according to Beijing local time (UTC + 8). The arrival height was set to 100 m above the ground. Meteorological data were obtained from the National Oceanic and Atmospheric Administration (ftp://arlftp.arlhq.noaa.gov/pub/archives/gdas0p5/, accessed on 6 January 2021) with a grid resolution of 0.25° × 0.25°. The threshold value was the average VOC concentration during the observation (Beijing 105.4 ppb, Baoding 97.1 ppb, Shanghai 91.1 ppb). The total number of trajectories was 1008 at each site.

### 2.6. Observation-Based Model

The observation-based model (OBM) was used in this study in combination with the Master Chemical Mechanism (v3.3.1; http://mcm.leeds.ac.uk/MCM/, accessed on 10 January 2021), a near-explicit mechanism describing the oxidation reactions of 146 primary VOCs and the latest inorganic chemistry data from the International Union of Pure and Applied Chemistry evaluation [51]. The OBM has been widely used to identify photochemical reactivity and photochemical products in various environments [52]. Hourly concentrations of the observed VOCs and four trace gases (CO, NO, NO_2,_ and SO_2_) and hourly meteorological parameters (temperature and relative humidity) were used as input data. The instantaneous concentration of VOCs was converted to hourly concentrations according to the linear regressions with CO, following the method by Yang et al. [53]. The OBM assesses the sensitivity of O_3_ photochemical production by calculating the relative incremental reactivity and altering the concentrations of its precursors without requiring detailed or accurate knowledge about these emissions [54]:

(8) $$RIR\left(X\right)=\frac{\left[P_{O_{3}}\left(X\right)-P_{O_{3}}\left(X-△X\right)\right]/P_{O_{3}}\left(X\right)}{△S\left(X\right)/S\left(X\right)}$$

where X is a precursor of O_3_, ΔX is the change in the concentration of X, P(O_3_) represents the net O_3_ production rate, S(X) is the measured concentration of precursor X, and ΔS(X)/S(X) represents the relative change in S(X), which was 20% in this study.

### 2.7. Human Health Risk Assessment

The USEPA proposed a method that uses the ambient mass concentration of air pollutants as an exposure evaluation parameter. The health risk of VOCs is divided into non-carcinogenic and carcinogenic risks, which are represented by the hazard quotient (HQ) and the lifetime carcinogenic risk (R), respectively [55]. An HQ less than 1 indicates no significant non-carcinogenic risk for adults, and an R-value less than 1 × 10^−6^ suggests an acceptable carcinogenic risk [38]. The specific calculation is shown in the Supplemental Material File.

## 3. Results and Discussion

### 3.1. Chemical Characteristics of Volatile Organic Compounds

**Figure 2:**
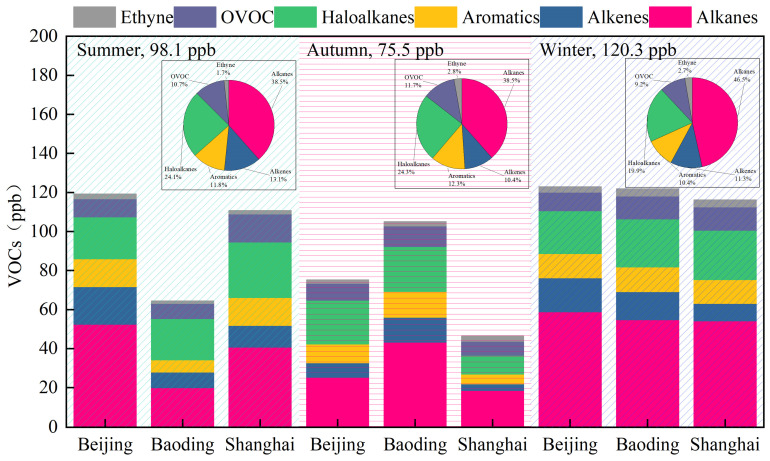
Concentrations and chemical characteristics of VOCs in different seasons during the observation period.

The concentrations of VOCs were the highest in winter (120.3 ± 61.5 ppb) among the three seasons assessed, followed by summer (98.1 ± 50.8 ppb) and autumn (75.5 ± 33.4 ppb), as shown in Figure 2. However, total VOCs were similar in the winter. In Baoding, VOC concentrations in the winter were significantly higher than those in the summer and autumn. VOC concentrations in the winter were 1.9 times those in the summer in Baoding. Baoding is a city of heavy industry, and the increase in industrial and heating emissions in the winter has led to an increase in VOC concentrations. VOC concentrations in the winter were close to those in the summer in Beijing and Shanghai. High temperatures and high solar radiation lead to higher solvent volatilization and plant-related VOC emissions in the summer. VOC concentrations in the autumn were the lowest of the three seasons.

**Figure 3:**
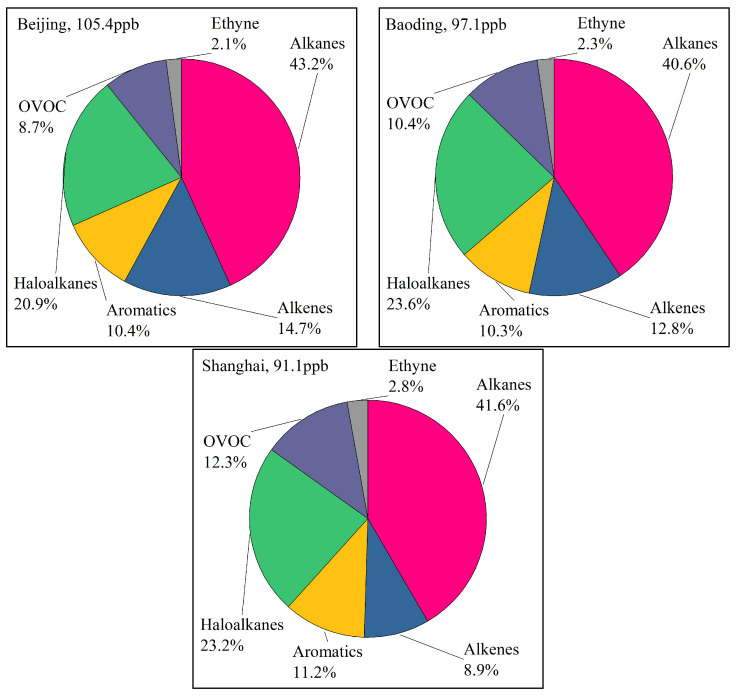
Chemical characteristics of VOCs observed in the three cities.

Alkanes were the dominant VOC species in all seasons, especially winter, exceeding 40% of the total (see Figure 3). The concentrations of haloalkanes were the second highest, and their proportion among the other VOCs decreased in the winter. The concentrations of alkenes were highest in the summer, and those of OVOCs and aromatics were highest in the autumn. The concentration of alkenes was higher in Beijing, and that of OVOCs was higher in Shanghai. The aromatic concentrations were at similar proportions in all three cities. VOC concentrations were highest in Beijing (105.4 ± 52.1 ppb), followed by Baoding (97.1 ± 47.5 ppb) and Shanghai (91.1 ± 41.3 ppb). The VOC concentrations in Beijing were 1.09 and 1.16 times those in Baoding and Shanghai, respectively. There were few differences in VOC concentrations throughout the year among the three cities. The differences in VOCs among cities were related to air pollutant emissions, sampling locations, and meteorological conditions.

Table 1 provides a comparison of the monitoring results from this study with previous observations of VOC species. The ethane, ethylene, propane, and acetylene concentrations in Shanghai were higher than those in previous reports. The concentrations of toluene and benzene in Shanghai were lower, and those of ethane and propane in Beijing were higher than those in previous studies. The ethylene, acetylene, and toluene concentrations in Beijing are similar to previously reported levels. The concentrations of VOC species in Baoding were higher than those in previous studies. Although the COVID-19 pandemic lockdown had a certain influence on industrial production in China, the concentration of VOC species did not show an obvious decreasing trend.

### 3.2. The Ozone Formation Potential of Volatile Organic Compounds

**Figure 4:**
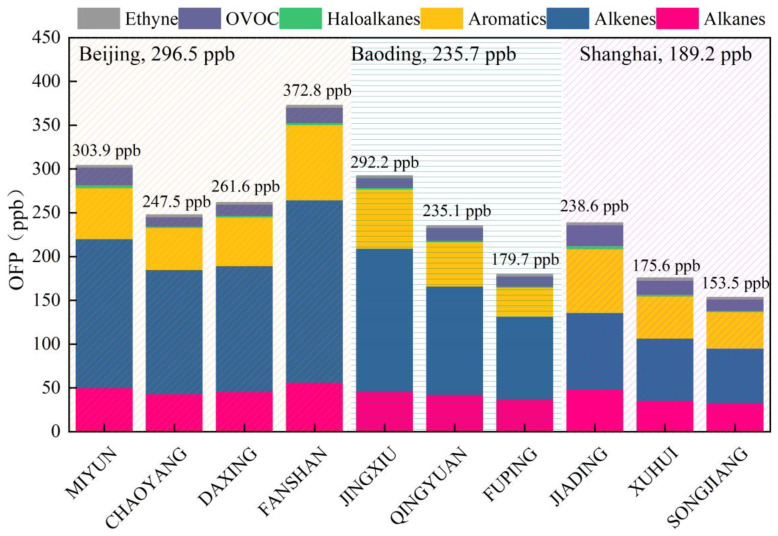
OFPs of VOC species at all sampling sites.

The OFP values of VOC species at the sampling sites were calculated (see Figure 4). Both VOCs and VOC OFP were at their maximum in Beijing. The concentrations of VOCs in Baoding and Shanghai were similar, but the OFP values were significantly lower in Shanghai than in Baoding. Alkenes were the most reactive species of VOCs in all cities, accounting for 56.0%, 53.7%, and 39.4% of the OFP in Beijing, Baoding, and Shanghai, respectively. Aromatics were the second most reactive species of VOCs, accounting for 20.7%, 21.0%, and 28.3% of the OFP in Beijing, Baoding, and Shanghai, respectively.

Notably, the OFP values of VOCs at Fangshan, a suburban site near a petrochemical plant, were the highest in Beijing. The OFP of VOCs at the urban Jingxiu site were the highest in Baoding. The OFPs of VOCs at Jiading, a background site, were the highest among sites in Shanghai. The top 10 VOC species with regard to OFP were identified and are shown in Table 2. The most reactive species were alkenes and aromatics, particularly ethene, propene, 1,3,5-trimethylbenzene, and m/p-xylene. The emission sources of these species should be strictly controlled.

### 3.3. Potential Source Areas of Volatile Organic Compounds

**Figure 5:**
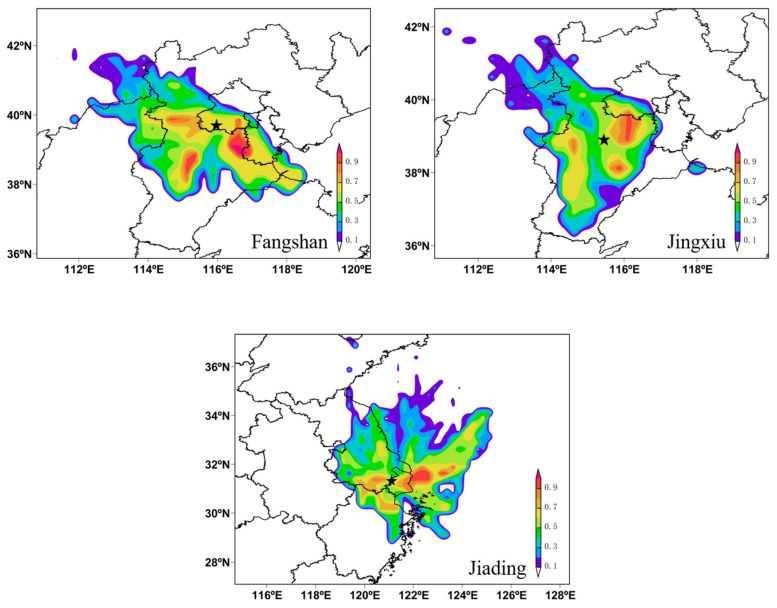
Potential source areas of VOCs for the Fangshan, Jingxiu, and Jiading sites, the five-pointed star referred to the sampling sites.

The Fangshan, Jingxiu, and Jiading sites were selected for source area analysis due to their high VOC concentrations and OFP values. The potential source areas of VOCs for the three cities were simulated, as shown in Figure 5. Three main potential source areas of VOCs for Fangshan were identified: the southeast region along the border of Beijing, Tianjin, and Hebei; the southwest region along the Taihang Mountains; and the western region. Two main potential source areas of VOCs were identified for Jingxiu: the northeast region near Beijing and the southeast region in Hebei. The potential source areas of VOCs for Jiading were located around the site and at sea. VOCs can be transported to and from the sea via airflow and ship emissions.

### 3.4. Source Apportionment of Volatile Organic Compounds

**Figure 6:**
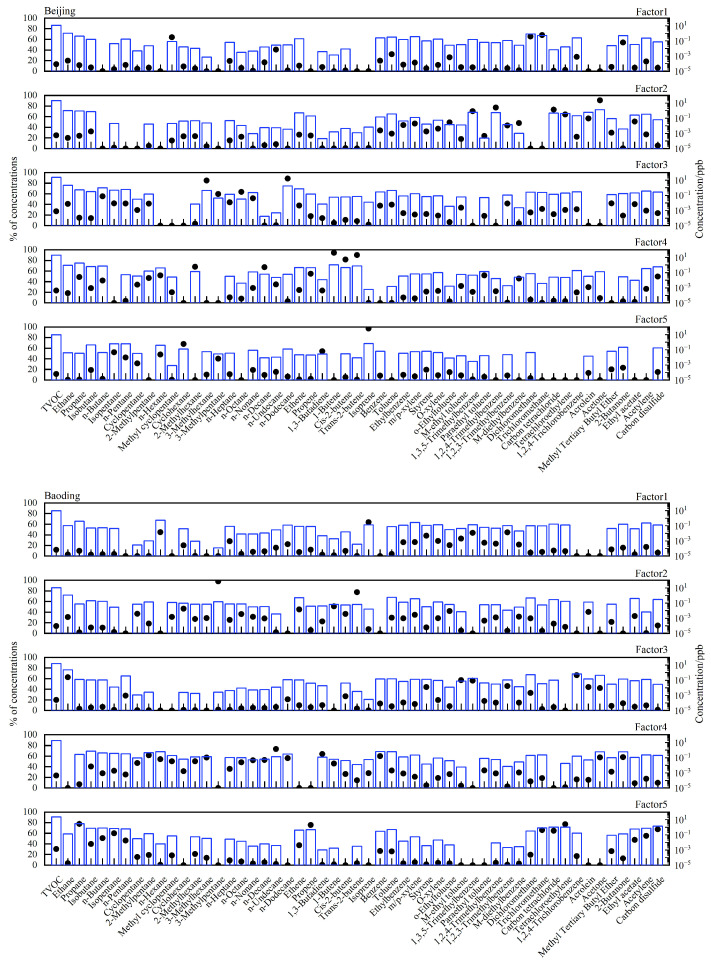

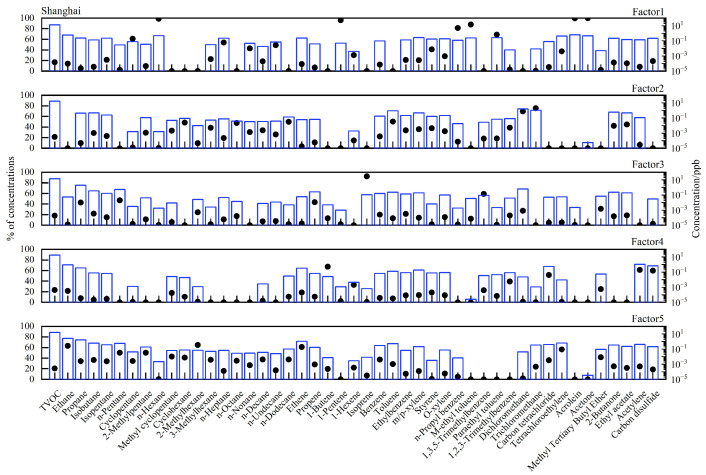
Five source profiles (bars) and contribution percentages (dots) representing each source factor were resolved using the PMF model.

We did not analyze species with a concentration below the MDL more than 50% of the time or with a significantly low signal-to-noise ratio [2,12]. After screening, 53 compounds in Beijing and Baoding and 47 compounds in Shanghai were selected. Five sources (vehicular exhaust, industrial manufacturing, solvent utilization, fuel combustion, and biogenic VOCs) were identified using the PMF model. Modeled source profiles and the relative contributions of individual sources to each species analyzed are shown in Figure 6.

In the source profiles for Beijing, the first source was characterized by significant amounts of methyl cyclopentane, n-undecane toluene, and 2-butanone, which are representative of industrial manufacturing [57]. The second source was characterized by high concentrations of carbon tetrachloride, tetrachloroethylene, and acetone, which are widely used as solvents [58]. The third source was associated with high concentrations of acetylene and alkane, such as isopentane, n-octane, and n-dodecane, which are major species in vehicular emissions [59]. The fourth source profile was rich in 1-butene, propane, and 2-methylhexane, tracers of fuel combustion [60]. The fifth source represented 97% of the total isoprene, considered the most important biogenic hydrocarbon [61].

In the source profiles for Baoding, the first source was characterized by a high concentration of isoprene(biogenic). The second source was characterized by significant amounts of 3-methylpentane, trans-2-butene, and 1-butene, which are representative of fuel combustion. The third source was associated with high concentrations of 1,2,4-trichlorobenzene and acetone, widely used as solvents. The fourth source profile was rich in benzene, toluene, n-undecane, and n-nonane, major species emitted from industrial manufacturing. The fifth source was characterized by high concentrations of acetylene, propane, and propene, tracers of vehicular exhaust.

In the source profiles for Shanghai, the first source was characterized by significant amounts of acetone, n-propyl benzene, and tetrachloroethylene, which are widely used as solvents. The second source profile was rich in dichloromethane, trichloromethane, toluene, and n-dodecane, major species emitted from industrial manufacturing. The third source represented 92% of the total isoprene, considered the most important biogenic hydrocarbon. The fourth source was characterized by high concentrations of 1-butene and 1-hexene, which are representative of fuel combustion. The fifth source was associated with high concentrations of methyl tertiary butyl ether, ethene, and ethane, major species emitted in vehicular exhaust.

**Figure 7:**
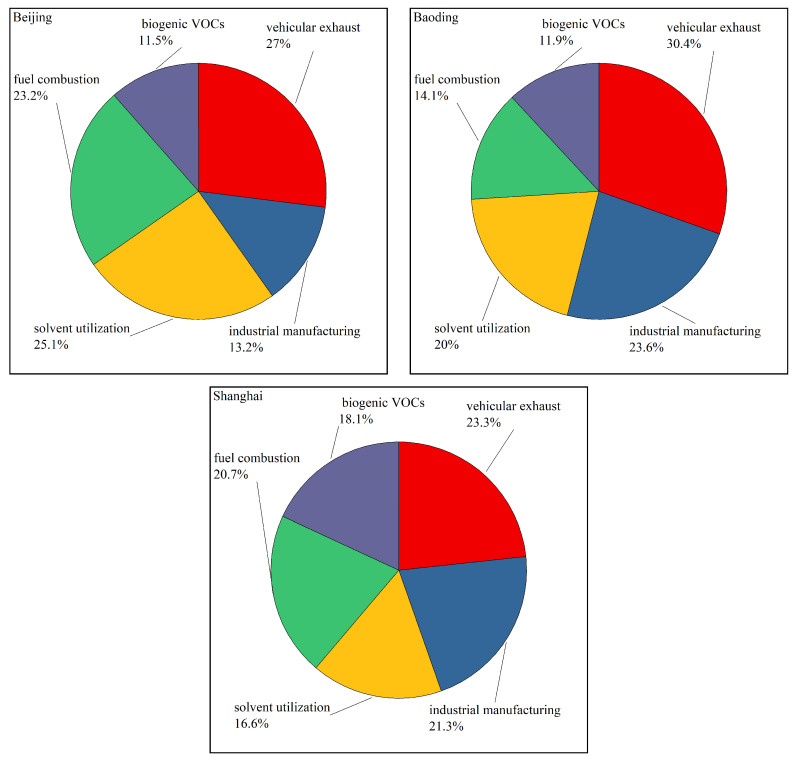
Source apportionment results for VOCs in the three cities.

Figure 7 illustrates the percentage source contributions during the sampling period in the three cities. Vehicular exhaust was the largest contributor in all three cities, accounting for 27.0%, 30.4%, and 23.3% of VOCs in Beijing, Baoding, and Shanghai, respectively. Industrial manufacturing was the second largest contributor in Baoding (23.6%) and Shanghai (21.3%), and solvent utilization was the second largest contributor in Beijing (25.1%). Fuel combustion was the third largest contributor in Beijing (23.2%) and Shanghai (20.7%), and solvent utilization was the third largest contributor in Baoding (20.0%). Biogenic sources of VOCs were also important, accounting for 11.5%, 11.9%, and 18.1% of VOCs in Beijing, Baoding, and Shanghai, respectively.

### 3.5. Empirical Kinetic Modeling Approach

**Figure 8:**
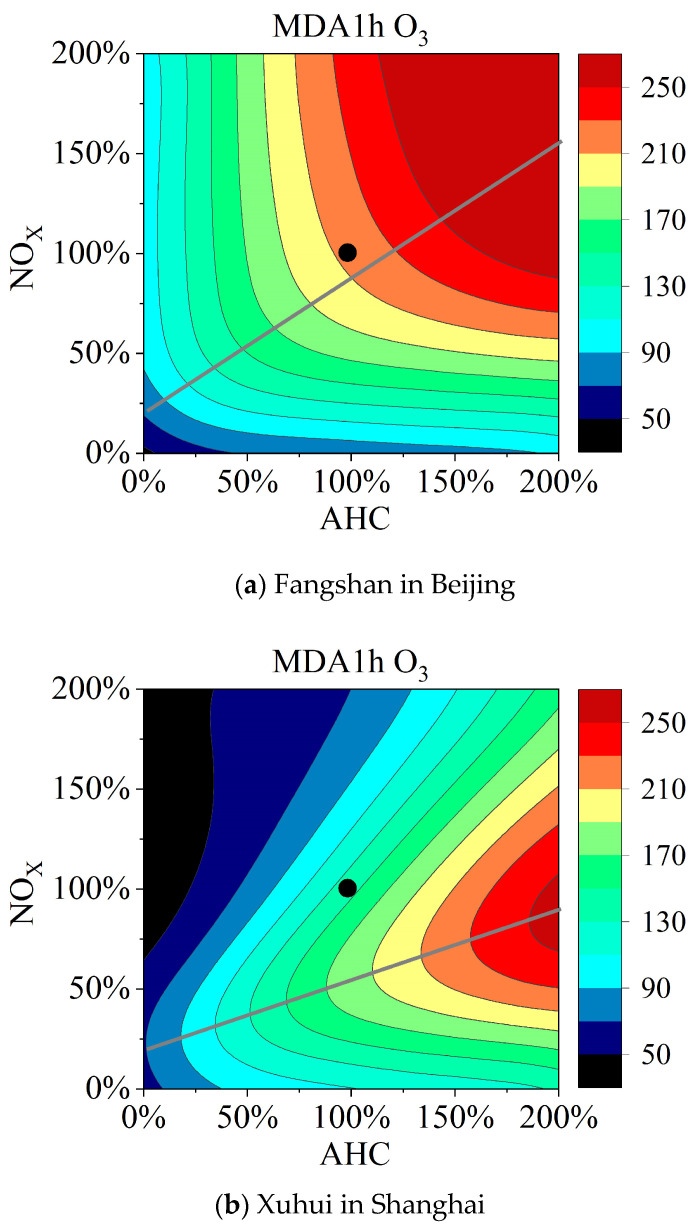
EKMA graphs for suburban Beijing and urban Shanghai. The black dot represents the base scenario, and the gray line represents the ridgeline of the EKMA curve. The AHC represents anthropogenic VOCs. The MDA 1 h O_3_ represents the daily maximum 1 h average O_3_ concentration.

Meteorological data for the Fangshan and Xuhui sites were obtained from the China Meteorological Station Data Sharing Service System. Thus, the empirical kinetic modeling approach (EKMA) curves for those two sites in the summer period were simulated using the OBM model, as shown in Figure 8. During the sampling period in summer, the average temperature and relative humidity were 30.4 °C and 60% in Fangshan and 31.5 °C and 71% in Xuhui. The EKMA plot was split into two areas by a ridgeline denoting the local maxima in the rate of O_3_ formation. The upper–left and lower–right areas represent O_3_ formation under VOC-limited and NOx-limited conditions, respectively. The base scenario point for the Fangshan site is located near the ridgeline, indicating a VOCs- and NOx-limited condition. The base scenario point for the Xuhui site is located in the upper-left area, indicating VOCs limitation. Fangshan is a suburban site, and Xuhui is an urban site. Previous studies have reported that urban and suburban areas in China were under VOC-limited and both VOC- and NOx-limited conditions [8,62], respectively.

### 3.6. Health Risk Assessment of Volatile Organic Compounds

**Figure 9:**
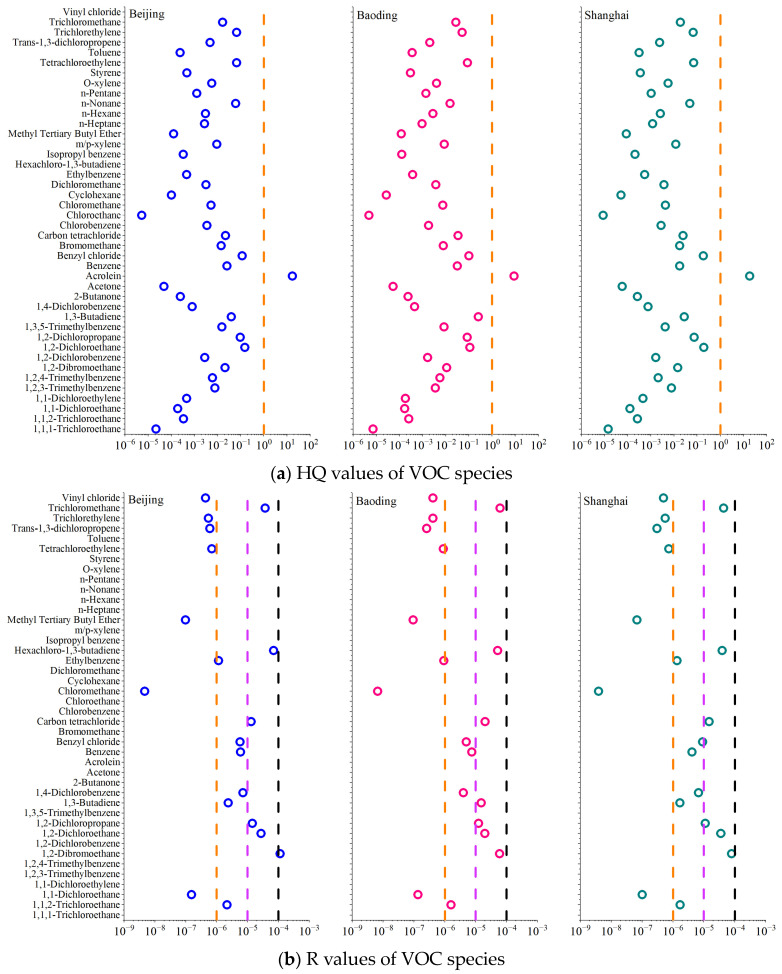
Non-carcinogenic and carcinogenic risks of VOC species in the three cities.

Forty-four species were targeted for health risk assessment, and their non-carcinogenic and carcinogenic risks are presented in Figure 9. The USEPA states that pollutants with an HQ of less than 1 pose no significant non-carcinogenic risk to adults. In this study, the average HQ values of the selected VOC species ranged from 5.3 × 10^−6^ to 16.9 × 10^−6^ in Beijing, 4.8 × 10^−6^ to 8.9 × 10^−6^ in Baoding, and 8.8 × 10^−6^ to 18.3 × 10^−6^ in Shanghai. Acrolein was the only substance with an average HQ value greater than 1, indicating significant non-carcinogenic risk. VOC species with carcinogenic risks of >10^−4^, 10^−5^ to 10^−4^, 10^−5^ to 10^−6,^ and <10^−6^ are classified as definite, probable, possible, and negligible risks, respectively [63]. In this study, the average R-value for the selected VOC species ranged from 4.7 × 10^−9^ to 1.1 × 10^−4^ in Beijing, from 6.7 × 10^−9^ to 6.2 × 10^−5^ in Baoding, and from 3.9 × 10^−9^ to 7.9 × 10^−5^ in Shanghai. In Beijing, 1,2-dibromoethane had an R-value of 1.1 × 10^−4^, posing a definite carcinogenic risk. Five, seven, and six VOC species posed probable carcinogenic risks in Beijing, Baoding, and Shanghai, respectively. Six, four, and six VOC species posed possible carcinogenic risks in Beijing, Baoding, and Shanghai, respectively. Among these species, hexachloro-1,3-butadiene, trichloromethane, 1,2-dichloroethane, and carbon tetrachloride posed high carcinogenic risks in all three cities.

## 4. Conclusions

In this study, field observations of CO, NO, NO_2_, O_3,_ and VOCs were conducted in three megacities in China: Beijing, Baoding, and Shanghai. VOC concentrations were highest in Beijing (105.4 ± 52.1 ppb), followed by Baoding (97.1 ± 47.5 ppb) and Shanghai (91.1 ± 41.3 ppb). VOC concentrations were highest in winter (120.3 ± 61.5 ppb) among the three seasons assessed, followed by summer (98.1 ± 50.8 ppb) and autumn (75.5 ± 33.4 ppb). Alkanes were the dominant species in all three cities, with concentrations exceeding 40%.

Alkenes were the most reactive VOC species in all three cities, accounting for 56.0%, 53.7%, and 39.4% of the OFP in Beijing, Baoding, and Shanghai, respectively. Aromatics were the second most reactive VOC species in all cities, accounting for 20.7%, 21.0%, and 28.3% of the OFP in Beijing, Baoding, and Shanghai, respectively. Most reactive species were alkenes and aromatics, particularly ethene, propene, 1,3,5-trimethylbenzene, and m/p-xylene.

Vehicular exhaust was the largest VOC source in all three cities, accounting for 27.0%, 30.4%, and 23.3% of VOCs in Beijing, Baoding, and Shanghai, respectively. Industrial manufacturing was the second largest contributor in Baoding (23.6%) and Shanghai (21.3%), and solvent utilization was the second largest contributor in Beijing (25.1%). Biogenic VOCs were also important, accounting for 11.5%, 11.9%, and 18.1% of VOCs in Beijing, Baoding, and Shanghai, respectively.

The EKMA approach indicated that O_3_ formation at the Fangshan site was limited by both VOCs and NOx, while that at the Xuhui site was limited by VOCs. Acrolein was the only substance with an average HQ value greater than 1, indicating a significant non-carcinogenic risk. In Beijing, 1,2-dibromoethane had an R-value of 1.1 × 10^−4^, posing a definite carcinogenic risk. Hexachloro-1,3-butadiene, trichloromethane, 1,2-dichloroethane, and carbon tetrachloride posed high carcinogenic risks in all three cities.

## Figures and Tables

**Table 1:** Comparison of the monitoring results for VOC species between the present study and previous reports.

Reference	Sampling Period	Sampling Site	Site Category	Monitoring Method	Ethane	Ethylene	Propane	Acetylene	Toluene	Benzene
Dai et al. (2010) [33]	2007–2010	Shanghai	Urban	Manual	—	—	4.81	—	4.70	1.81
Zheng et al. (2019) [56]	Autumn 2016	Shanghai	Urban	Online	2.22	1.52	3.59	1.17	5.04	0.70
Zheng et al. (2019) [56]	Autumn 2016	Shanghai	Suburban	Online	3.01	0.99	4.22	0.03	0.96	0.44
Zhang et al. (2020) [7]	7 April to 25 September 2018	Shanghai	Suburban	Online	1.26	1.56	2.93	0.73	1.87	—
This study	Summer to Winter 2019	Shanghai	Urban	Manual	5.98	2.60	6.87	2.89	2.28	0.93
Zhang et al. (2020) [7]	Autumn 2016	Beijing	Urban	Online	3.42	2.13	2.85	0.68	2.00	4.74
Zhang et al. (2020) [7]	Winter 2016	Beijing	Urban	Online	4.60	2.43	6.70	0.26	1.82	6.04
Zhang et al. (2020) [7]	Spring 2017	Beijing	Urban	Online	1.93	0.59	2.33	0.51	1.17	5.41
Zhang et al. (2020) [7]	Summer 2017	Beijing	Urban	Online	2.33	0.57	2.65	0.90	1.34	6.99
Shi et al. (2020) [39]	December 2016 to January 2017	Beijing	Urban	Online	—	12.07	—	8.98	3.63	3.27
This study	Summer to Winter 2019	Beijing	Urban	Manual	7.37	2.59	7.21	2.27	1.81	1.14
Wang et al. (2021) [28]	May to September 2019	Baoding	Urban	Online	3.98	1.51	2.19	0.37	0.58	0.31
This study	Summer to Winter 2019	Baoding	Urban	Manual	5.01	2.16	5.85	2.27	2.61	1.94

**Table 2:** Top 10 VOC species with the highest OFP values in the three cities.

Cities	VOCs	OFP
Beijing	Ethene	32.6
	Trans-2-butene	29.5
	1-Butene	18.8
	Propene	18.4
	Cis-2-butene	17.4
	1,3,5-Trimethylbenzene	13.3
	1-Pentene	13.1
	Isoprene	12.2
	1-Hexene	10.2
	m/p-xylene	9.7
Baoding	Ethene	26.5
	Cis-2-pentene	16.4
	1,3-Butadiene	16.1
	Propene	15.1
	Trans-2-butene	14.0
	1-Pentene	11.6
	Toluene	11.3
	m/p-xylene	9.1
	1,3,5-Trimethylbenzene	8.6
	1-Butene	7.8
Shanghai	Ethene	24.6
	m/p-xylene	12.7
	Propene	12.4
	Toluene	9.9
	Cis-2-pentene	8.0
	1-Pentene	7.5
	Acrolein	7.1
	1,2,3-Trimethylbenzene	6.9
	1-Hexene	6.4
	O-xylene	5.9

## Data Availability

The data are available on request from the corresponding author.

## Supplementary Materials

The following supporting information can be downloaded at: https://www.mdpi.com/article/10.3390/toxics11080651/s1, Table S1: RfC and IUR values of selected VOC species in this study [64].
