# Supplementary material for: Ambient Volatile Organic Compound Characterization, Source Apportionment, and Risk Assessment in Three Megacities of China in 2019
Source: Toxics. 2023 Jul 27;11(8):651. doi: 10.3390/toxics11080651 (PMC10458435; doi:10.3390/toxics11080651)
Supplement: Supplementary file 1 [file toxics-11-00651-s001.zip › toxics-2409586-supplementary.pdf]

Ambient Volatile Organic Compound Characterization, Source Apportionment, and Risk Assessment in Three Megacities of China in 2019

Zhanshan Wang[1, +,*], Puzhen Zhang[1, +], Libo Pan[1, +], Qian Yan[1], Zhigang Li[1], Xiaoqian Li[1], Chen Guo[1], Xiaojing Zhu[1], Yuanyuan Xie[2], Yongjie Wei[1]

[1] State Key Laboratory of Environmental Criteria and Risk Assessment, Chinese Research Academy of Environmental Sciences, Beijing, China

[2] Foreign Environmental Cooperation Centre, Ministry of Ecology and Environment, Beijing 100035, China

2. Methodology

2.7 Human health risk assessment

(1) Exposure concentration (EC)

$$EC = C \times \frac{ET \times EF \times ED}{AT} \qquad (9)$$

where C represents the mass concentration of a given VOC species (μg m^{-3}), ET represents the exposure time (h d^{-1}), EF represents the exposure frequency (d y^{-1}), ED represents the exposure duration (y), and AT represents the averaging time (h). According to the USEPA Integrated Risk Information System (IRIS) and the Exposure Factors Handbook of the Chinese Population (for adults) (MEP, 2013), ET, EF, ED, and AT were set to 3.7 h d^{-1}, 365 d y^{-1}, 74.8 y, and 24 h, respectively.

(2) Non-carcinogenic risk

The hazard index (HI) is used to estimate non-carcinogenic risk and is the sum of the HQ values of VOC species, as shown in Eq. (10). HQ represents the ratio of EC to the inhalation reference concentration (RfC, mg/m^3), as shown in Eq. (11).

$$HI = \sum_{i=1}^{n} HQ_i \qquad (10)$$

$$HQ = \frac{EC}{RfC} \qquad (11)$$

(3) Carcinogenic risk

Carcinogenic risk assessment is conducted using the cumulative cancer risk (CR), which is the

sum of the lifetime carcinogenic risk (R) of VOC species, as shown in Eq. (12). Carcinogenic risk (R) is the product of the exposure concentration and inhalation unit risk (IUR), as shown in Eq. (13).

$$CR = \sum_{i=1}^{n} R_i \qquad (12)$$

$$\mathrm{R} = \mathrm{EC} \times \mathrm{IUR} \qquad (13)$$

In this study, the RfC and IUR of each VOC species were set to the values recommended by the IRIS, the Agency for Toxic Substances and Disease Registry, the Provisional Peer-Reviewed Toxicity Values of the IRIS, the California Environmental Protection Agency, and the Health Effects Assessment Summary Tables, as noted in Table 1. Forty-four target species were selected for health risk assessment based on the following principles: species classified by the International Agency for Research on Cancer as groups 1, 2A, or 2B, representing key toxic compounds and species frequently reported in the literature to allow for comparison.

Table 1. RfC and IUR values of selected VOC species in this study

Species	RfC (μg m^{-3})	IUR (μg m^{-3})
1,1,2-Trichloroethane	400	0.000016
1,1-Dichloroethane	500	0.0000016
1,2-dibromoethane	9	0.0006
1,2-Dichloroethane	7	0.000026
1,2-Dichloropropane	4	0.000037
1,3-Butadiene	2	0.00003
1,4-Dichlorobenzene	800	0.000011
Benzene	30	0.0000078
Benzyl chloride	1	0.000049
Carbon tetrachloride	100	0.000006
Trichloromethane	98	0.000023
Chloromethane	90	0.00000001
Ethylbenzene	1000	0.0000025
Methyl Tertiary Butyl Ether	3000	0.00000026
Tetrachloroethylene	40	0.00000026
trans-1,3-Dichloropropene	31.8	0.000004

Trichlorethylene	2	0.0000041
Hexachloro-1,3-butadiene		0.000022
Carbon tetrachloride		0.000006
Vinyl chloride		0.0000088
1,1,1-Trichloroethane	5000	
1,1-Dichloroethylene	200	
1,2,3-Trimethylbenzene	60	
1,2,4-Trimethylbenzene	60	
1,2-Dichlorobenzene	200	
1,3,5-Trimethylbenzene	60	
Acetone	31000	
Acrolein	0.02	
Bromomethane	5	
Chlorobenzene	50	
Chloroethane	10000	
Cyclohexane	6000	
dichloromethane	600	
Isopropyl benzene	400	
m/p-Xylene	100	
2-Butanone	5000	
n-Heptane	400	
n-Hexane	700	
n-Nonane	20	
n-Pentane	1000	
o-Xylene	100	
Styrene	1000	
Carbon tetrachloride	100	
Toluene	5000	

References

Ministry of Environmental Protection of the People's Republic of China (MEP), 2013. Exposure Factors Handbook of Chinese Population. Chinese Environmental Science Press, Beijing, China, pp. 798–801.
